# Structural changes and influencing factors of human resource allocation for oral health in China

**DOI:** 10.1371/journal.pone.0324454

**Published:** 2025-05-22

**Authors:** Hong Tan

**Affiliations:** Department of Periodontal Mucosal Disease, The Affiliated Stomatology Hospital of Southwest Medical University, Oral & Maxillofacial Reconstruction and Regeneration of Luzhou Key Laboratory, Luzhou, Sichuan, China; Fooyin University, TAIWAN

## Abstract

**Objective:**

In the context of the implementation of the Action Program for a Healthy Dentition in China, to analyze the structural changes and influencing factors of human resource allocation for oral health in China, and to provide a scientific basis for promoting the development of human resources for oral health.

**Methods:**

Structural change degree analysis (by analyzing the structural change of the components of things to reflect the overall characteristics of the structural change of things) and grey relational analysis (by determining the degree of similarity of the geometric shapes of the reference series and a number of comparison series to determine whether they are closely related) were used to analyze the structural changes and influencing factors of human resources for oral health from 2017 to 2022.

**Results:**

The age of human resources for oral health in China is mainly 25 ~ 34 years old and 35 ~ 44 years old, with a proportion of 37.30% and 33.30%, respectively, in 2022. The number of years of work experience is mainly 10–19 years, with a proportion of 26.80% in 2022. Educational qualifications are mainly at the college level, with a proportion of 41.20% in 2022. Professional and technical qualifications are mainly at the division/assistant level, with a proportion of 52.30% in 2022. The structural change values for ages 45 ~ 54 and 60 and over are negative overall, with a negative trend. The structural change degree of age reaches 13.60% in 2020, which is a dynamic structural change. The structural change values for work experience of 10 years or more are negative overall, with a negative trend. The structural change values for post-secondary and undergraduate education are positive, with a positive trend. The structural change degree of education fluctuates range from 2.60% to 5.20%. The structural change values for associate and intermediate levels are negative, with a negative trend. The structural change degree of professional and technical qualifications reaches its highest value of 8.80% in 2021. The most influential factor was per capita health expenditure, with a relational of 0.787, followed by per capita disposable income, with a relational of 0.682, and thirdly, per capita GDP, with a relational of 0.667.

**Conclusion:**

Age of 45 ~ 54 and years of work experience of more than 10 years show an overall negative trend. College and undergraduate education show a positive trend. Per capita health expenditure and per capita disposable income are the main factors influencing the allocation of human resources for oral health. Therefore, the administration should formulate inclined policies to continuously strengthen the introduction and cultivation of human resources for oral health, and all medical institutions should emphasize the academic education and continuing education of dentists, so as to comprehensively promote the development of oral health in China.

## Introduction

The World Health Organization defines oral health as teeth that are clean, free of decay and pain, and gums that are normally colored and free of bleeding. Oral health is the foundation of general health, and the combination of good oral hygiene habits and regular professional oral health care can maintain oral health, promote general health, and improve the quality of life. Improving oral health depends on the rational allocation of oral health resources. The allocation of oral health resources is a key indicator of the level of oral health care services in a country or region, and human resources for oral health are the most dynamic of oral health resources. With the gradual increase in people’s awareness of oral health, the demand for oral health services is increasing, which has brought newer requirements for oral health resource allocation [1]. Oral health resource allocation is influenced by population, economic and social factors. Health resource allocation is usually based on the number of people per 1,000 population as an allocation standard, and the larger the population size, the higher the corresponding health resource allocation. Health resource allocation is also influenced by economic factors, and the higher the level of economic development, the higher the corresponding health resource allocation. The influence of social factors on the health resource allocation is mainly manifested in the extent of the burden of health expenditure, the perception of and demand for medical services, and so on. By analyzing the structural proportions and changes in the components of health resources, it is possible to find out the development direction of the structural changes in health resources, which is conducive to optimizing the structural proportions of health resources and promoting the overall development of health resources. The World Health Organization member countries have proposed an oral health strategy to achieve universal oral health coverage by 2030 [2]. In 2019, the Notice of the General Office of the National Health Commission on the Issuance of the Action Program for a Healthy Dentition (2019–2025) (National Health Office Disease Control Letter [2019] No. 118) proposed to coordinate the allocation of oral health resources, improve the oral health service system, and comprehensively improve the level of oral health. To continuously improve the oral health service system [3], it is necessary to improve and optimize the allocation of human resource for oral health, identify the major influencing factors of human resource for oral health, and promote the mobility of human resource for oral health, in order to better meet people’s oral health needs [4].

At present, international studies on oral health resources include needs-based human resource planning for oral health in Sierra Leone [5], the situation of human resources for oral health in the African region [6], and the situation of the health workforce in addressing oral health inequities [7], and so on. In relevant international studies, it has been found that human resources for oral health in the African region are scarce, and that the global distribution of dentists, as well as the overall global oral health workforce, is inequitable, and other issues. Specifically, studies on oral health resources in China can be divided into the following three sections. First, studies on oral health resource allocation and service utilization, mainly including the statistical analysis of current oral health care and dental education resources in China [8], the utilization of oral health services and the economic burden of oral diseases in China [9], the pattern of utilization of oral health services for preschool children in Beijing [10], and the factors associated with the utilization of oral health services among adults and older adults in China [11]. Second, studies on the oral health status of special populations, mainly including the oral health status and knowledge of only and non-only children in China [12], the oral health and dental status of epileptic patients in rural China [13], and the oral health behavior and oral health service utilization of cancer patients in China [14]. Third, studies on oral health knowledge and policies, mainly including oral health knowledge, beliefs and practices among community-dwelling older adults in Shanghai [15], oral health policies to address the burden of early childhood caries [16], and improving the oral health of older adults for healthy aging [17]. At present, studies on oral health resources in China mainly focus on the utilization of oral health services and the oral health status of special populations, and the overall studies on human resource allocation for oral health in China are still somewhat inadequate. What is the situation of human resource allocation for oral health in China, what are the structural changes in human resources for oral health, and what are the factors affecting the allocation of human resources for oral health are all worthy of in-depth study. By using structural change degree analysis to find out the internal composition and structural changes of human resource allocation for oral health, and by using grey relational analysis to find out the influencing factors of human resource allocation for oral health in China, it is conducive to improve the human resource allocation for oral health, better improve the oral health service system, and promote the development of the action program for a healthy dentition.

In order to improve and optimize the allocation of human resources for oral health in China, this study used structural change degree analysis and grey relational analysis to analyze the structural changes and influencing factors of human resources for oral health in China from 2017 to 2022, and to provide a scientific basis for promoting the development of human resources for oral health.

## Materials and methods

### Data sources

Data on human resources for oral health in China were obtained from the China Health Statistical Yearbook from 2018 to 2023, and human resources for oral health refer to those who practice under the category of oral licensed (assistant) physicians, excluding those who are actually engaged in management work. The scope of the statistics includes data from 31 provincial-level administrative regions in mainland China, and does not include data from Hong Kong, Macau, and Taiwan due to inconsistencies in statistical calibers.

### Indicators

Labor resources are the basic part of human resources, which includes the sum of persons engaged in labor work of different age, gender, years of work experience, educational qualifications and professional and technical qualifications. Human resources for oral health are the sum of personnel actually engaged in oral health services according to the health needs of the local population, therefore, age, years of work experience, educational qualifications, and professional and technical qualifications were selected as evaluation indicators of structural change [18].

Combining the availability of data and related studies, the allocation of oral health resources can be influenced by population, economic, and social factors [19]. In terms of population, a larger population generally implies a greater allocation of health resources, the dependency ratio measures the dependency borne by the workforce per capita, and the level of urbanization promotes the construction of oral health infrastructure, so the number of resident population (10,000), the proportion of population aged 65 years or older (%), the dependency ratio (%), and the level of urbanization (%) were selected as influencing factors in terms of population. In terms of economy, per capita GDP can measure the level of regional economic development, and per capita disposable income can measure the population’s ability to pay, so per capita GDP (RMB) and per capita disposable income (RMB) are selected as the influencing factors of economy. On the social side, the ratio of health expenditure to GDP and per capita health expenditure can reflect the degree of burden of health expenditure on society and individuals, so the per capita health expenditure (RMB) and the ratio of the health expenditure to the GDP (%) were selected as the influencing factors on the social side.

### Research methods

Structural change degree analysis and grey relational analysis were used to analyze the structural changes and influencing factors of human resources for oral health in China from 2017 to 2022.

### Structural change degree analysis

Structural change degree analysis is to reflect the overall characteristics of structural change of things by analyzing the structural change of each component within things, including structural change value, structural change degree and structural change contribution rate. The structural change value is the difference between the end-of-period and the beginning-of-period values of the component ratios for a given period, where a value greater than 0 indicates a positive trend, and vice versa for a negative trend. The structural change degree is the sum of the absolute values of the structural change values, where the larger the value, the greater the structural change degree. The structural change contribution rate is the absolute value of the structural change value of each component ratio as a proportion of the structural change degree, and the larger the value, the greater the impact on structural change.

### Grey relational analysis

Grey relational analysis is used to determine the degree of similarity of the geometric shapes of the reference series and a number of comparison series to determine whether they are closely related [20], the greater the degree of similarity, the greater the relationship between the corresponding series, and vice versa, the smaller the relationship. Grey system theory takes the grey system with the characteristics of “small sample, poor information and uncertainty” as the research object, and realizes the exploration of the grey system evolution law and the prediction of the development trend through the mining and analysis of known information. Human resource allocation for oral health is affected by a variety of factors such as population, economy and society, which is a typical grey system with part of the information known and part of the information unknown, so it can be analyzed by using the grey relational analysis. First, the eight independent variables are used as the comparison series and the human resources for oral health are used as the reference series. Second, the initial value method is used to perform dimensionless processing, and the discrimination coefficient ρ is taken as 0.5 in accordance with the usual situation. Finally, the relational degree and the ranking of the influencing factors can be calculated [21].

## Results

### Composition of age, years of work experience, educational qualification, and professional and technical qualifications of human resources for oral health

**Fig 1:**
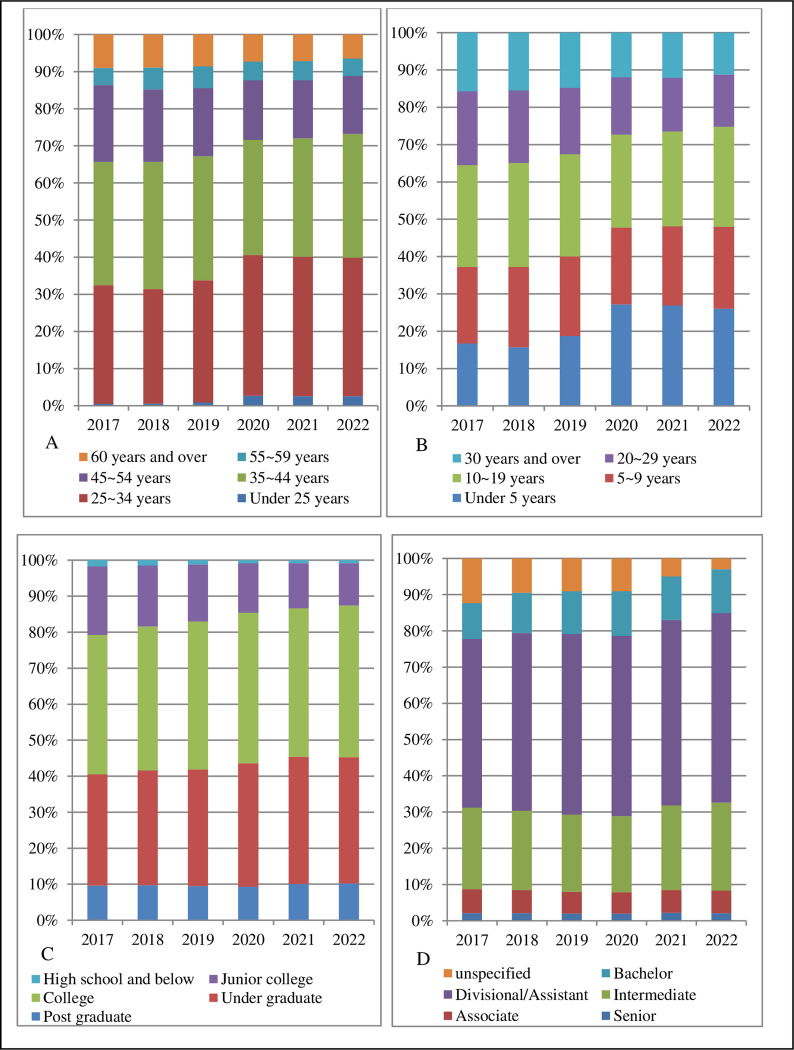
Composition of age (A), years of work experience (B), educational qualification (C), and professional and technical qualifications (D) of human resources for oral health (%).

The age of human resources for oral health in China is dominated by 25 ~ 34 years old and 35 ~ 44 years old, accounting for about one-third of the total, respectively. Under the age of 25 years old is the smallest percentage at 2.60% in 2022, but the overall percentage has increased, indicating some replenishment of human resources for oral health. Years of work experience is dominated by 10 ~ 19 years, accounting for about a quarter of the total. The proportion of years of work experience under 5 years has increased overall, reaching 27.20% in 2020, and then decreased after 2020, indicating that there is a significant increase in the young force of human resources for oral health, but a shortage of the backbone of the force. The educational qualification of human resources for oral health in China is dominated by the college level, with the proportion of 38.70% in 2017 increasing to 42.10% in 2022; followed by the undergraduate level, with the proportion of 30.90% in 2017 increasing to 35.10% in 2022, indicating that the educational qualification structure of human resources for oral health has been continuously optimized and the overall quality of personnel has been continuously strengthened. The overall proportion of postgraduate level is within 10.20%, indicating that there is a shortage of highly educated personnel. Professional and technical qualifications are dominated by divisional/assistant level, with the proportion of 46.60% in 2017 increasing to 52.30% in 2022, indicating an overall low level of professional and technical qualifications. This is followed by intermediate level, with a proportion of 22.50% in 2017 increasing to 24.30% in 2022, and the overall proportion of senior professional level is within 10% (Fig 1).

### Structural change value, structural change degree and structural change contribution rate of age of human resources for oral health

The structural change values for ages 45 ~ 54 and 60 years and over are negative overall, indicating a negative trend, while those for ages under 25 years old are positive overall, indicating a positive trend. The structural change degree of age fluctuates widely, reaching 13.60% in 2020, with an active structural change. The structural change contribution rate of 25 ~ 44 years old is more than 46%, which is characterized by repeated fluctuations, while the structural change contribution rate of less than 25 years old is always small, but reaches a maximum value of 13.24% in 2020 (Table 1).

**Table 1:** Structural change value, structural change degree and structural change contribution rate of age of human resources for oral health.

Year	Structural change value						Structural change degree (%)	Structural change contribution rate (%)					
	Under 25 years	25 ~ 34 years	35 ~ 44 years	45 ~ 54 years	55 ~ 59 years	60 years and over	Under 25 years	25 ~ 34 years	35 ~ 44 years	45 ~ 54 years	55 ~ 59 years	60 years and over	
2017	0.001	-0.014	0.007	-0.001	0.001	0.006	3.00	3.33	46.67	23.33	3.33	3.33	20.00
2018	0.001	-0.012	0.011	-0.012	0.013	-0.001	5.00	2.00	24.00	22.00	24.00	26.00	2.00
2019	0.003	0.021	-0.009	-0.012	0.000	-0.003	4.80	6.25	43.75	18.75	25.00	0.00	6.25
2020	0.018	0.050	-0.024	-0.023	-0.008	-0.013	13.60	13.24	36.76	17.65	16.91	5.88	9.56
2021	-0.001	-0.004	0.009	-0.004	0.001	-0.001	2.00	5.00	20.00	45.00	20.00	5.00	5.00
2022	0.000	-0.002	0.014	0.000	-0.005	-0.007	2.80	0.00	7.14	50.00	0.00	17.86	25.00

### Structural change value, structural change degree and structural change contribution rate of years of work experience of human resources for oral health

The structural change values for more than 10 years of work experience are negative overall, indicating a negative trend. The structural change values for under 5 years of work experience are positive overall, indicating a positive trend. The structural change degree of years of work experience shows an increase and then a decrease and then an increase, with a maximum of 17.00% in 2020, which is an active structural change. The structural change contribution rate of under 5 years is more active, accounting for more than 20% of the total, except in 2021 (Table 2).

**Table 2:** Structural change value, structural change degree and structural change contribution rate of years of work experience of human resources for oral health.

Year	Structural change value					Structural change degree (%)	Structural change contribution rate (%)				
	Under 5 years	5 ~ 9 years	10 ~ 19 years	20 ~ 29 years	30 years and over	Under 5 years	5 ~ 9 years	10 ~ 19 years	20 ~ 29 years	30 years and over	
2017	0.008	0.005	-0.002	-0.006	-0.005	2.60	30.77	19.23	7.69	23.08	19.23
2018	-0.010	0.010	0.006	-0.004	-0.002	3.20	31.25	31.25	18.75	12.50	6.25
2019	0.030	-0.002	-0.005	-0.016	-0.007	6.00	50.00	3.33	8.33	26.67	11.67
2020	0.085	-0.007	-0.025	-0.025	-0.028	17.00	50.00	4.12	14.71	14.71	16.47
2021	-0.003	0.006	0.005	-0.009	0.001	2.40	12.50	25.00	20.83	37.50	4.17
2022	-0.009	0.008	0.014	-0.004	-0.009	4.40	20.45	18.18	31.82	9.09	20.45

### Structural change value, structural change degree and structural change contribution rate of educational qualifications of human resources for oral health

The structural change value for junior colleges and below is negative, indicating a negative trend, while the structural change value for colleges and undergraduates is positive overall, indicating a positive trend. The overall structural change degree of educational qualifications is low, fluctuating between 2.60% and 5.20%, with an average degree of active structural change. The structural change contribution rate of junior colleges is more than 30%, while the structural change contribution rate of high schools and below is less than 10% (Table 3).

**Table 3:** Structural change value, structural change degree and structural change contribution rate of educational qualifications of human resources for oral health.

Year	Structural change value					Structural change degree (%)	Structural change contribution rate (%)				
	Post graduate	Under graduate	College	Junior college	High school and below	Post graduate	Under graduate	College	Junior college		High school and below
2017	0.004	0.009	0.000	-0.011	-0.002	2.60	15.38	34.62	0.00	42.31	7.69
2018	0.001	0.010	0.013	-0.022	-0.002	4.80	2.08	20.83	27.08	45.83	4.17
2019	-0.002	0.004	0.012	-0.011	-0.003	3.20	6.25	12.50	37.50	34.38	9.38
2020	-0.002	0.020	0.006	-0.021	-0.003	5.20	3.85	38.46	11.54	40.38	5.77
2021	0.008	0.01	-0.006	-0.011	-0.001	3.60	22.22	27.78	16.67	30.56	2.78
2022	0.001	-0.002	0.009	-0.008	0.000	2.00	5.00	10.00	45.00	40.00	0.00

### Structure change value, structure change degree and structure change contribution rate of professional and technical qualifications of human resources for oral health

The structural change values for the associate and intermediate levels are negative overall, indicating a negative trend, while the structural change values for the bachelor and senior levels are positive overall, indicating a positive trend. The structural change degree of professional and technical qualifications shows repeated fluctuations and reaches a maximum value of 8.80% in 2021, with active structural change. The structural change contribution rate of the senior and associate levels is within 14%, while the structural change contribution rate of the intermediate levels and division/assistant levels is above 40% (Table 4).

**Table 4:** Structure change value, structure change degree and structure change contribution rate of professional and technical qualifications of human resources for oral health.

Year	Structural change value						Structural change degree (%)	Structural change contribution rate (%)					
	Senior	Associate	Intermediate	Divisional/ Assistant	Bachelor	unspecified	Senior	Associate	Intermediate	Divisional/ Assistant	Bachelor	unspecified	
2017	-0.001	-0.002	-0.010	-0.005	-0.003	0.021	4.20	2.38	4.76	23.81	11.90	7.14	50.00
2018	0.000	-0.003	-0.005	0.024	0.012	-0.028	7.20	0.00	4.17	6.94	33.33	16.67	38.89
2019	-0.001	-0.003	-0.007	0.008	0.007	-0.004	3.00	3.33	10.00	23.33	26.67	23.33	13.33
2020	0.000	-0.001	-0.003	-0.001	0.005	0.000	1.00	0.00	10.00	30.00	10.00	50.00	0.00
2021	0.002	0.004	0.023	0.015	-0.003	-0.041	8.80	2.27	4.55	26.14	17.05	3.41	46.59
2022	-0.001	-0.001	0.010	0.011	0.001	-0.020	4.40	2.27	2.27	22.73	25.00	2.27	45.45

### Grey relational analysis of influencing factors of human resources for oral health

According to the results of the grey relational analysis, except for the number of resident population, which has a relational degree of 0.490, the relational degrees of the other seven indicators are all greater than 0.523, indicating a strong relation between human resources for oral health and the influencing factors. The largest influencing factor on the human resource allocation for oral health was the per capita health expenditure, with a relational degree of 0.800, followed by per capita disposable income, with a relational degree of 0.673, and the third factor was the per capita GDP, with a relational degree of 0.667. The relational degree of factors influencing on the human resource allocation for oral health was ranked in descending order as the per capita health expenditure, per capita disposable income, per capita GDP, proportion of population aged 65 years or older, dependency ratio, ratio of health expenditure to the GDP, level of urbanization, and the number of resident population (Table 5).

**Table 5:** Grey relational analysis of influencing factors of human resources for oral health.

Year	Number of resident population (10,000)	Proportion of population aged 65 years or older (%)	Dependency ratio	Level of urbanization	Per capita GDP (RMB)	Per capita disposable income (RMB)	Per capita health expenditure (RMB)	Ratio of the health expenditure to the GDP (%)
2017	0.335	0.476	0.424	0.369	0.569	0.569	0.726	0.383
2018	0.451	0.634	0.574	0.487	0.645	0.672	0.803	0.519
2019	0.677	0.832	0.860	0.699	0.805	0.778	0.855	0.703
2020	0.708	0.833	0.998	0.750	0.674	0.781	0.932	1.000
2021	0.432	0.582	0.544	0.467	0.698	0.662	0.671	0.424
2022	0.339	0.509	0.413	0.368	0.612	0.574	0.814	0.384
Relational degree	0.490	0.644	0.635	0.523	0.667	0.673	0.800	0.569
Rank by relational degree	8	4	5	7	3	2	1	6

## Discussion

In 2017–2022, the number of human resources for oral health in China under the age of 25 years old and with less than 5 years of work experience continues to increase, which shows that with the concept of “oral health, whole body health” continuously deepened, the policy attaches importance to the inclusion of oral health in the health planning, which promotes the development of oral health resources in China [22]. With the growth of social economy and people’s attention to oral health, pulling the overall development of oral health resources. It is important to note that the age of 45 ~ 54 years old and the years of work experience of more than 10 years show an overall negative trend, which indicates that the backbone of human resources for oral health is insufficient, and there is a phenomenon of loss of human resources [23]. We believe that there are two reasons for this. On the one hand, with a large number of young forces entering the oral industry, older personnel are gradually withdrawing from the oral industry [24], which objectively results in a gradual decrease in the number of years of work experience of more than 10 years, with a significant negative trend, and the oral industry is characterized by an overall younger, shorter period of time in the profession, and a shortage of backbone forces. On the other hand, it is constrained by factors such as more limited development of the oral career, inadequate remuneration, job burnout with lower job satisfaction, etc [25]. In recent years, there has been a phenomenon of many oral personnel jumping ship to other industries, resulting in the problem of the loss of oral personnel [26]. Therefore, it is necessary to continuously strengthen the training of human resources for oral health, absorb more medical students to join the dental industry, so as to promote the development of oral health in China. It is also necessary to strengthen the career development planning of human resources for oral health and to gradually raise the level of remuneration and benefits, so as to reduce the wastage of oral health personnel [27]. It is also necessary to promote and regulate the multi-point practice of dentists, promote the reasonable flow of regional dental talents, and comprehensively improve the level of oral health.

From 2017 to 2022, the educational level of human resources for oral health in China has gradually improved, and the college and undergraduate levels have shown a positive trend, which indicates that the educational structure of human resources for oral health has been continuously optimized, and the overall quality of personnel has been continuously strengthened, which helps to meet the people’s growing demand of people for oral health medical services. We believe that there are two reasons for this. On the one hand, with the popularization of higher education and the massive expansion of oral medicine colleges and universities, the educational level of oral health personnel has been gradually improved, with the number of personnel with less than junior college education gradually decreasing and the number of personnel with college education or higher gradually increasing. With the development of the social economy and the importance attached to education, more and more people choose to invest in themselves to improve their educational level in order to get better development space and remuneration, which further promotes the upgrading of oral health personnel’s educational qualifications [28]. It is worth paying attention to the current stage is still mainly college level, especially the private oral health care institutions of health personnel with lower education, human resources for oral health as a whole education level still has a lot of room for improvement. On the other hand, the overall percentage of postgraduate level of education is within 10.20%, indicating that there is still a shortage of highly educated personnel, which affects the development of high-quality oral health resources. Highly educated personnel have longer training time and high academic pressure, resulting in relatively slow growth of highly educated personnel. Highly educated personnel have higher demands for career development opportunities and remuneration packages, and are relatively more concentrated in large cities with developed economies, convenient transportation and large populations, resulting in uneven distribution of high-quality oral health resources and obvious differences among regions [29]. Therefore, it is necessary to attach importance to the academic education of human resources for oral health in terms of policy, encourage more medical students to enter the oral health profession, and continuously improve the allocation of human resources for oral health. Medical institutions should also attach importance to the continuing education of oral health practitioners, and increase the opportunities for practitioners to upgrade their academic qualifications and pursue continuing education [30]. It is also necessary to increase investment in equipment and facilities for oral services in medical institutions, improve the capacity of primary medical and health care institutions to provide oral medical services, promote the importance of oral health care for children and regular oral check-ups, and comprehensively improve the level of oral health services.

From 2017 to 2022, the professional and technical qualifications of human resources for oral health in China were mainly at the divisional/assistant level, and the overall level of professional and technical qualifications was low, which resulted in an overall shortage of oral health care and limited the development of the overall level of human resources for oral health. First, the overall educational level of human resources for oral health is relatively low, and it is more difficult to obtain professional and technical qualifications at the next level, coupled with fewer opportunities for relevant continuing education and further training, and slow updating of professional knowledge, it becomes relatively difficult to pass the examination. Second, with the increasing demand for oral health care services, many departments are short of nursing staff, which does not allow them to do the four-handed operation of medical and nursing care, and does not do a good job of health education for patients, doctors have to spend a lot of time and energy on patient communication and health education, which is not conducive to improving the professional knowledge and skills of doctors. It is worth paying attention to the negative trend of the associate and intermediate levels, this group has both a certain work experience and a certain business ability, and plays the role of medical backbone in the whole oral health care industry, and its negative trend leads to the shortage of experts and discipline leaders with a certain degree of influence, which affects the construction of the oral health care personnel team [31]. Therefore, it is important to emphasize the promotion of the professional and technical qualifications of human resources for oral health and to provide appropriate opportunities for continuing education and training [32]. It is also necessary to strengthen the nursing staff of oral health care institutions so that doctors can focus more on improving their professional and technical skills [33]. The development of the oral medicine industry should adhere to the reform of the professional and technical qualification and title structure that combines functional positioning and talent requirements, support the tilting of intermediate and senior positions toward oral medicine practitioners [34], and gradually promote the simultaneous upgrading of years of work experience, educational qualifications, and professional and technical qualifications of human resources for oral health [35].

The relational degree of 0.800 for per capita health expenditure and 0.673 for per capita disposable income are the top two influencing factors, indicating that per capita health expenditure and per capita disposable income are the main factors influencing the allocation of human resources for oral health. On the one hand, economic development improves the economic base of tax revenues, which promotes greater government investment in health care resources and improves the supply capacity of health care services. Economic development promotes the growth of health expenditure [36], which in turn has a greater impact on the growth of health resources. Economic development also promotes the population’s demand for health care resources. Economic development leads to an increase in people’s income, and people’s investment in health care services increases, thus promoting the development of health care services. On the other hand, as people’s awareness of health care increases, the demand for oral health services increases, and the increase in per capita disposable income has created a situation where people are able to spend more of their income on the expenditure of oral health services, which in turn affects the allocation of oral health resources [37]. The level of urbanization and the number of resident population are the seventh and eighth factors influencing the allocation of human resources for oral health, indicating that the mere growth of population and the increase in the level of urbanization alone cannot significantly improve the allocation of human resources for oral health. Oral health services have traditionally not been considered as necessary health care services and require a certain level of health expenditure, resulting in oral health services not being health services for all, but rather health services with a certain income base and willingness to pay [38]. Therefore, it is necessary to consider socio-economic development as the key to promoting regional economic development in order to stabilize and increase health expenditure and people’s disposable income [39]. It is also necessary to strengthen the concept of “oral health, whole body health” in the action program for a healthy dentition to raise people’s awareness and level of oral health care.

## Limitations

Although we used structural change degree analysis and grey relational analysis to analyze the structural changes and influencing factors of human resources for oral health in China from 2017 to 2022, this study still has some limitations. First, the allocation of human resources for oral health includes aspects such as equity, efficiency, and service utilization, which are not covered in this study. Second, this study is an overall study of the human resource allocation for oral health, and does not cover aspects such as regional differences. Third, human resources for oral health are also affected by health policies, which were not included in this study.

## Conclusion

In order to promote the development of human resources for oral health, we used the structural change degree analysis and grey relational analysis to analyze the structural changes and influencing factors of human resources for oral health in China from 2017 to 2022. The study found that the age of human resources for oral health in China is mainly 25 ~ 34 years old and 35 ~ 44 years old. The number of years of work experience is mainly 10–19 years. Educational qualifications are mainly at the college level, with a proportion of 41.20% in 2021. Professional and technical qualifications are mainly at the division/assistant level, with a proportion of 51.20% in 2021. The structural change values for ages 45 ~ 54 and 60 and over are negative overall, with a negative trend. The structural change values for work experience of 10 years or more are negative overall, with a negative trend. The structural change values for post-secondary and undergraduate education are positive, with a positive trend. The structural change degree of education fluctuates range from 2.60% to 5.20%. The structural change values for associate and intermediate levels are negative, with a negative trend. The structural change degree of professional and technical qualifications reaches its highest value of 8.80% in 2021. The most influential factor on the human resource allocation for oral health was per capita health expenditure, with a relational of 0.800, followed by per capita disposable income, with a relational of 0.673. We suggest that the administration should formulate inclined policies to continuously strengthen the introduction and cultivation of human resources for oral health, further promote and standardize the multi-practice practice of dentists, and promote the reasonable mobility of regional dental talents, so as to promote the development of oral health in China. All medical institutions should pay attention to the academic education and continuing education of dentists, increase the opportunities for physicians to improve their academic qualifications and continuing education, implement the promotion of professional and technical qualifications of human resources for oral health, strengthen the capacity building of oral health care services in primary health care institutions, and comprehensively improve the level of oral health care services. It is also necessary to maintain socio-economic development, increase investment in health expenditure, vigorously popularize knowledge of oral health care, and gradually improve the level of human resources for oral health.

## Supporting information

S1 Table Data on human resources for oral health in China, 2017–2022. (XLSX)
